# Two Cases of Partial Paralysis of the Posterior Extremities

**Published:** 1880-10

**Authors:** William Soula


### TWO CASES OF PARTIAL PARALYSIS OF THE POSTERIOR EXTREMITIES.

By William Soula.

The histories and post-mortem findings in these two cases differ, but there is similarity enough to class them together.

Case I.—A bay mare, *æt.* 6, weight about eleven hundred pounds. This mare, to all appearance, was perfectly well up to the day of the attack, which was four days before death.

The first symptom noticed was, that the horse, while out on the road, suddenly became stiff in the posterior extremities. This stiffness was almost immediately followed by great loss of motor power, so that it was with difficulty that the mare could be got back to the stable. When she arrived at the stable, she could no longer support the weight of her body, and she was placed in a sling. The posterior extremities were very cold, while the general temperature was a little above normal. On both sides of the vertebral column, in the lumbar and sacral region, there was so much swelling, that a deep hollow was left in the medium line.

Tincture of belladonna and nux vomica were administered. The urine was drawn with a catheter, and found to be thick, and of a very dark color. The bowels were moved by an enema. The above treatment was continued during the next twenty four hours. During the day the mare passed water four times. At the end of the twenty-four hours the horse was eating and drinking well, and hope of a speedy recovery was entertained. The next day the symptoms remained unchanged. On the third day the mare would not eat, and became semi-comatose. The urine was now passed very frequently and in small quantities, being thick, and almost black in color. The temperature rose to 107° F. This increase of temperature may have been in part due to the constant straining. From this time the mare gradually sank, and died, comatose, on the morning of the fourth day, at four o'clock A.M. It might be well to state here, that the mare was in heat at the time she was attacked.

At three o'clock P. M., on the day of her death, a necropsy was held, in the presence of Dr. Porter. Rigor mortis was well marked. The mare was in good flesh.

Abdominal Cavity.—When the abdomen was opened two quarts of coagulated blood and bloody serum was found in the peritoneal sack. The liver, spleen, and intestines were normal. The kidneys were intensely congested; a little pus was visible in the pelves. They were in a state of commencing parenchymatous degeneration. All the tissues and muscles in the lumbar and sacral region were filled with extravasated blood and bloody serum.

THORACIC CAVITY.—Heart and lungs normal.

The brain and spinal cord were removed. The membranes and convexity of the brain were deeply congested, but no evidence of acute inflammation existed. The spinal cord and membranes were normal, not even congested.

CASE II.—This case was probably similar to the preceding in the start, although that part of the history is wanting. A bay mare, *æt.* 6, weight about eleven hundred pounds. When this case was first seen by me a number of weeks had elapsed since the onset of the disease. At the time the mare was first seen she was suffering from partial paralysis of the posterior extremities, and could only walk when well supported in a sling. At this time she was passing a small quantity of urine, and this at very short intervals. *Examination of Urine.* The urine was very dark in color; thick, and had a very offensive odor, no albumen. The microscopical examination gave pus, blood corpuscles, salts of the lime carbonates, oxalate of lime, considerable granular matter, and some granular bodies strongly resembling casts. The case was looked upon as one of partial paraplegia, congestion of the kidneys, and cystitis. The treatment in this case was dry cupping of the loins, hyoscyamus, and potass. bicarbonate internally, to act upon the kidneys and bladder; damiana as a nerve tonic to the spinal cord, with moderate exercise while supported in the sling. For several weeks the mare steadily improved until she could almost stand without any aid from the sling. The urine became clear, and was passed normally. The bad odor also disappeared. She gained strength and fed well, when, to the surprise of all, the mare suddenly sank and died.

The necropsy was held on the day of her death, Dr. Porter being present. Rigor mortis was well marked, mare fairly well nourished.

THORACIC CAVITY.—Heart normal; lungs oedematous.

ABDOMINAL CAVITY.—In the cardiac extremity of the stomach two bots were found clinging firmly to the mucus membrane. At the pyloric extremity of the stomach numerous spots of ulceration were found in the mucus membrane. The balance of the alimentary tract was normal; liver and spleen normal.

KIDNEYS.—Both kidneys were deeply congested, and pus was found in the pelves. The kidneys, although congested, looked white and fatty throughout the cortical portion.

BLADDER.—The bladder was opened and found to be entirely filled with a mass looking like clay. This mass weighed several pounds, was soft, not hard like a calculus. It became very hard after exposure to the air. A sample of this mass was examined by Professor Louis H. Laudy and found to contain lime carbonate in abundance. The urine had evidently made its way along one side of the mass to the urethra. After this mass had been removed from the bladder, the mucus coat was found congested, extensively ulcerated, and in some places almost gangrenous.

The uterus and ovaries were normal.

After carefully going over these two cases, a number of interesting points will be noticed, some of which I will now refer to.

The fact has been observed, that these attacks of partial posterior paralysis are more frequent in mares than in geldings; the proportion being five to one. Mares also are most likely to die. Another important fact is, that after a mare has stood in the stable for a few days without exercise, and is then taken out in a cold wet rain, especially if in heat, she is very apt to have one of these attacks and die.

Some authors have been inclined to regard these cases as spinal meningitis, but the result of the necropsy in this first case is proof to the contrary, and points more toward the kidney as the seat of the trouble. Just how this almost total loss of power is to be explained, will need more cases to clearly elucidate.

If it can be established that the period of heat acts as a predisposing cause, then by great care at this time, or perhaps by spaying, the number of these cases may be diminished, and the lives of many valuable horses spared.

Can a plan of treatment be instituted which will tend to secure recovery?

If the disease is located in the kidneys, as these two cases apparently were, then the most rational plan of treatment would be that of counter-irritation to the loins, in the form of dry cupping or otherwise, with the internal administration of non-irritating diuretics

Now that the dangers that may result from chronic cystitis, in the form of deposits in the bladder of inorganic salts, is known and appreciated, this need not be looked upon as a serious drawback to recovery. In case they pass through the acute attack and become chronic, the bladder should be washed out every day with some bland disinfecting solution. By so doing, the bladder may be kept clean and the deposition of organic matter prevented.

Another question arises in connection with these cases: Is kidney disease a common thing among horses, and if so, is it generally amenable to treatment?

